# Fetal Circulatory Variation in an Acute Incident Causing Bradycardia

**DOI:** 10.1155/2014/820318

**Published:** 2014-12-18

**Authors:** Safak Olgan, Mehmet Sakinci, Murat Ozekinci, Nasuh Utku Dogan, Erkan Cagliyan, Sabahattin Altunyurt

**Affiliations:** ^1^Department of Obstetrics and Gynecology, Akdeniz University Faculty of Medicine, 07059 Antalya, Turkey; ^2^Department of Obstetrics and Gynecology, Dokuz Eylul University Faculty of Medicine, 35340 Izmir, Turkey

## Abstract

Umbilical artery\vein, middle cerebral artery, and ductus venosus Doppler velocimetry were performed at 33 weeks of gestation in the settings of an intrauterine growth restricted fetus during a heart rate deceleration. Interestingly, we recorded a sudden onset redistribution of fetal blood flow with fetal bradycardia. Spontaneous normalization of waveforms was observed once fetal heart rate returned to normal. Our case provides evidence to circulatory variation of a human fetus resulting from an acute incident causing bradycardia.

## 1. Introduction

Hypoxemia was known to alter fetal cardiac functions. The major alterations can be summarized as the decrease in cardiac output, increase in arterial pressure and associated afterload, and major redistribution of the blood flow by selective peripheral vasoconstriction [1]. Doppler velocimetry reflects the hypoxemic status of the fetus, and decrease in oxygen saturation subsequently results in changes of fetal blood flow waveforms [2]. However, we neither know the circulatory changes that occur with an acute insult such as fetal bradycardia nor if there is a chance of spontaneous normalization as the primary insult ceases in an intrauterine growth restricted human fetus.

## 2. Case Presentation

**Figure 1:**
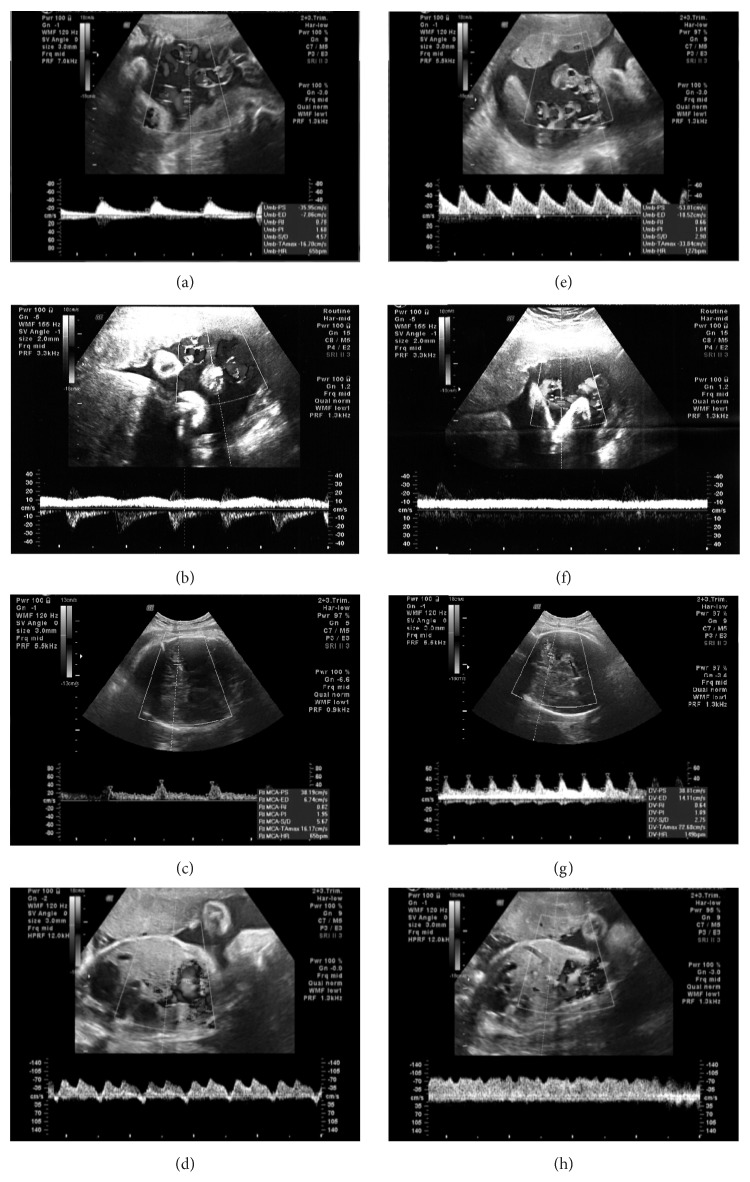
Umbilical artery (a), umbilical vein (b), middle cerebral artery (MCA) (c), and ductus venosus (d) Doppler velocimetry during the deceleration period and Doppler velocimetry of these vessels at normal heart rate, accordingly (e, f, g, h).

**Figure 2:**
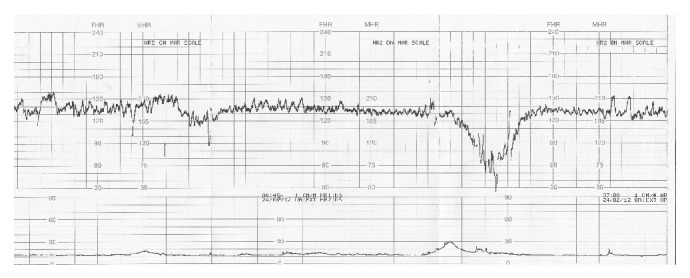
Cardiotocographic (CTG) monitoring revealed a late type of deceleration after a mild uterine contraction.

A 38-year-old multiparous woman at 33 weeks of gestation was referred to Dokuz Eylul University Hospital for evaluation of fetal intrauterine growth restriction (IUGR). Maternal vital signs were within normal limits at the time of admission. Cardiotocographic (CTG) monitoring revealed nonreassuring fetal heart rate tracing and few fetal movements. There were mild uterine contractions 20 minutes apart. A Voluson V730 Expert (GE Healthcare, Milwaukee, WI, USA) ultrasound machine was used for ultrasound examination. Fetal biometry was below 5th percentile, but amniotic fluid index was consistent with normal amniotic fluid volume (85 mm). Early in Doppler ultrasonography, we manually determined a uterine contraction and then coincidentally recorded the fetal Doppler velocimetry during a heart rate deceleration (70 beats/minute, 4 minutes). During this period, fetal umbilical artery blood flow diminished progressively till diastolic blood flow disappeared (Figure 1(a)). Umbilical vein was in single pulsating pattern (Figure 1(b)). Middle cerebral artery (MCA) consistent with brain sparing turned into high resistance pattern (Figure 1(c)). Interestingly, characteristic ductus venosus (DV) tracing, demonstrating marked prolongation of ventricular diastole with delayed onset of reversed A-wave, was recorded (Figure 1(d)). Spontaneous reversion of these waveforms to predeceleration phase was observed once fetal heart rate returned to normal (Figures 1(e), 1(f), 1(g), and 1(h)). The patient was hospitalized and initially monitored with continuous CTG. Shortly after the fetal monitoring, we detected a late type of deceleration for a second time (Figure 2). She was recommended bed rest and hydrated with 1000 mL of lactated Ringer solution. Additionally, maternal corticosteroid was given (two doses of intramuscular betamethasone 12 mg 24 hours apart) for fetal lung maturation. Through the follow-up in high risk obstetrical service for seven days, the fetus did not demonstrate any other sign of severe fetal distress on CTG, amniotic fluid index, biophysical profile score, or Doppler velocimetry. Finally, the patient underwent cesarean delivery for recurrent late decelerations at 34 weeks of gestation, which resulted in the birth of a low-birth-weight boy (1720 g, 5th–10th percentile); the 1-minute and 5-minute Apgar scores were 8 and 9, respectively. The infant's umbilical cord blood gas showed an arterial pH of 7.26.

## 3. Discussion

In the acute sequence of animal studies by closure of the umbilical vessels were found decreased heart rate, slightly increased stroke volume, and a constant cardiac output [3]. Similar to our case, Siristatidis et al. found increased resistance indices in the umbilical circulation as the oxygen saturation fell. They postulated that this may reflect a reaction by which the fetus keeps blood in its own vascular space, even if poorly oxygenated, and takes advantage of the higher affinity of fetal hemoglobin for oxygen [4]. Although the liver receives most of the well-oxygenated umbilical venous blood, like chronic hypoxemia, increased shunting through the DV during acute insult is an important defense mechanism to maintain oxygenation in the central circulation [5]. The proportion of umbilical blood passing through DV also increased in acute hypoxemia together with DV PI (pulsatility index) and augmented pulsations [3, 6]. Likewise, Gudmundsson et al. induced hypoxemia in ovine fetuses and observed correspondingly decreased end diastolic blood velocity in DV and pulsating pattern in the umbilical vein during fetal bradycardia [7]. Dealing with the venous circulation increasing pulsatility was found to be associated with failure of compensatory mechanisms through right heart failure due to an increased afterload and myocardial hypoxia [8]. We recorded a DV tracing, demonstrating a marked prolongation of ventricular diastole with a delayed onset of reversed A-wave together with a single pulsating umbilical vein comparable to the literature. Regarding the human fetuses, the only case was reported by Szunyogh and coworkers. They observed a transient type of atypical ductus venosus waveform during a fetal heart rate deceleration in labor accordingly [9]. Unlike chronic hypoxia, it was found that the fetuses with an already established brain-sparing flow show no further cerebral hyperperfusion in response to a superimposed hypoxemia [10]. Fu and Olofsson demonstrated not only a decrease of MCA PI in an acute sequence, but also that some fetuses respond to hypoxic stress with a marked increase of the PI [10]. Likewise, in our case, MCA pattern consistent with brain sparing turned into high resistance pattern during bradycardia.

Since the umbilical artery pH was 7.26 at birth, there was no evidence of chronic hypoxia in our case. It was known that Doppler waveforms are a function of (1) upstream pressure (cardiac output), (2) heart rate, (3) vessel diameter, length and compliance (fixed unless compressed by external force), and (4) downstream resistance (resistance to blood flow through the placental bed for the UV, resistance in the cerebral vasculature for the MCA) [11]. Subsequently, the changes in the umbilical artery and MCA could be attributed entirely to period length in our case. The end diastolic velocity will continue to slow until systole, so the PI naturally increases as the heart rate slows. We also believe that a contribution of hypoxia to this change cannot be excluded but cannot be assessed independently. Additionally, the peak systolic velocities are only mildly reduced during this phase, suggesting only a marginal drop in left ventricular pressure.

The reversal in DV A-wave cannot be explained by period length prolongation and is reflected in the monophasic umbilical vein waveform. This may reflect acute hypoxia causing reduced cardiac contractility. Reversal of flow in the A-wave is a recognized association of cardiac decompensation associated with chronic fetal hypoxemia, but as discussed above the cord blood gases do not confirm this. Moreover, the rapid recovery of a normal A-wave is difficult to explain. Since our case was an IUGR affected fetus, these findings might have different mechanisms in normal fetuses as proposed by the other studies [12–14].

To the best of our knowledge, this case is the first dealing with both arterial and venous circulatory changes of a human fetus during a fetal bradycardia. During the course of the bradycardia, we observed a swift change from fetal compensatory state to failure of compensation and finally to compensatory state again. Therefore, an acute incident might suddenly alter the current circulatory status of a fetus. Future studies are warranted in order to understand the clinical significance of this phenomenon.
